# Diversity of inland valleys and opportunities for agricultural development in Sierra Leone

**DOI:** 10.1371/journal.pone.0180059

**Published:** 2017-06-29

**Authors:** Elliott Ronald Dossou-Yovo, Idriss Baggie, Justin Fagnombo Djagba, Sander Jaap Zwart

**Affiliations:** 1 Geographic Information System and Remote Sensing Unit, Africa Rice Center (AfricaRice), Cotonou, Benin; 2 Rokupr Agricultural Research Centre (RARC), Sierra Leone Agricultural Research Institute (SLARI), Rokupr, Sierra Leone; TNO, NETHERLANDS

## Abstract

Inland valleys are becoming increasingly important agricultural production areas for rural households in sub-Saharan Africa due to their relative high and secure water availability and soil fertility. In addition, inland valleys are important as water buffer and biodiversity hot spots and they provide local communities with forest, forage, and fishing resources. As different inland-valley ecosystem functions may conflict with agricultural objectives, indiscriminate development should be avoided. This study aims to analyze the diversity of inland valleys in Sierra Leone and to develop guidelines for more precise interventions. Land use, biophysical and socio-economic data were analyzed on 257 inland valleys using spatial and multivariate techniques. Five cluster groups of inland valleys were identified: (i) semi-permanently flooded with high soil organic carbon (4.2%) and moderate available phosphorus (10.2 ppm), mostly under natural vegetation; (ii) semi-permanently flooded with low soil organic carbon (1.5%) and very low available phosphorus (3.1 ppm), abandoned by farmers; (iii) seasonally flooded with moderate soil organic carbon (3.1%) and low available phosphorus (8.3 ppm), used for rainfed rice and off-season vegetables produced without fertilizer application for household consumption and market; (iv) well drained with moderate soil organic carbon (3.8%) and moderate available phosphorus (10.0 ppm), used for rainfed rice and off-season vegetables produced with fertilizer application for household consumption and market; and (v) well drained with moderate soil organic carbon (3.6%) and moderate available phosphorus (11 ppm), used for household consumption without fertilizer application. Soil organic carbon, available phosphorus, hydrological regime, physical accessibility and market opportunity were the major factors affecting agricultural intensification of inland valleys. Opening up the areas in which inland valleys occur through improved roads and markets, and better water control through drainage infrastructures along with an integrated nutrient management would promote the sustainable agricultural use of inland valleys.

## Introduction

Inland valley ecosystems are estimated to cover about 3.6% of sub-Saharan Africa [1], corresponding to approximately 85 million ha [2]. Inland valleys are defined as the upper parts of river drainage systems, comprising the whole upland lowland continuum [3], from the rainfed uplands (pluvial) to rainfed, flooded and intensified lowlands in the valley bottom (fluxial), with the hydromorphic fringes (phreatic) as the (sloping) transition zone between them [4]. Inland valleys were not obvious ecosystems for agricultural production, and traditionally have not often been used for agriculture in sub-Saharan Africa [5, 6]. This is partly because inland valley bottoms are difficult to manage and are also often associated with water-borne diseases such as bilharzia (schistosomiasis—*Schistosoma haematobium* and *S*. *mansoni*), river blindness (onchocerciasis—*Wolbachia pipientis*), sleeping sickness (trypanosomiasis—*Trypanosoma brucei gambiense* and *T*. *brucei rhodesiense*) and malaria (e.g. *Plasmodium falciparum*, *P*. *malariae* and *P*. *ovale*) [7, 8]. Despite such challenges, inland valleys have increasingly been put under production by more recent generations. Global changes, such as population growth and climate change, provide new incentives for inland valley agricultural use [9]. With rich soils and year-round water and / or soil moisture availability, inland valleys provide smallholder farmers with opportunities to produce crops year-round, including the dry season and particularly during drought years, thereby mitigating food shortages from upland fields and improving farmers’ incomes [10, 11]. Various agronomic methods developed in inland valleys include expansion of the cultivated area by draining swampy valleys, increased frequency of cropping seasons, and use of agricultural inputs. Such methods have resulted in extension, intensification and / or diversification of agricultural use in these areas [12].

In addition, inland valleys deliver a range of associated ecosystem functions [5]. They are important for local flood and erosion control, water storage, nutrient retention and stabilization of the micro-climate, as well as for recreation and tourism, and for providing water, clay and sand for crafts and construction [9]. These environments provide important forest, wildlife and fisheries resources, and contribute to biological diversity as well as local cultural heritage [5, 13]. As different inland valley ecosystem functions may conflict with agricultural objectives, and because there are area-specific differences in development suitability and risks, indiscriminate development should be avoided [14]. Considering the current rate of inland valley conversion into sites of production and the diverse ecological, social and production functions that inland valleys fulfill, there is a need to provide guidelines for their future protection or use [15]. Such decision support requires a systematic classification and characterization of inland valleys by identifying the extent and diversity of their types and uses, while providing a better understanding of the physical (shape, climate, soils, hydrology), and socio-economic environments within which inland valleys occur. Additionally, understanding the diversity of inland valley users’ strategies may also help in developing guidelines for their future protection and sustainable use.

Although the characterization of inland valley agro-ecosystems has been discussed since the 1990s, most studies have focused on physical characterization [3, 16, 17, 18, 19]. Few studies have included socio-economic characteristics in the classification of inland valleys [20, 21, 15]. At present, characterization approaches combining biophysical, socio-economic and land-use factors have rarely been applied [15]. Additionally, little is known about the production systems, the patterns of diversity and their relationship with production objectives in smallholder farming systems in inland valleys [22]. This study combined biophysical, land use and socio-economic data including farmers’ production systems and production objective providing a more comprehensive socio-ecological classification of inland valleys than the existing ones.

We hypothesized that the diversity of biophysical characteristics of inland valleys and of the socio-economic attributes of their surrounding environments are determinants in farmers’ decision-making with respect to their uses (type, intensity and duration). The aims of the study were to: (i) identify the diversity of inland valleys and uses in the districts of Bo and Kenema, Sierra Leone; and (ii) determine the factors affecting farmers’ decisions with respect to their use of inland valleys.

## Materials and methods

### Study area

**Fig 1:**
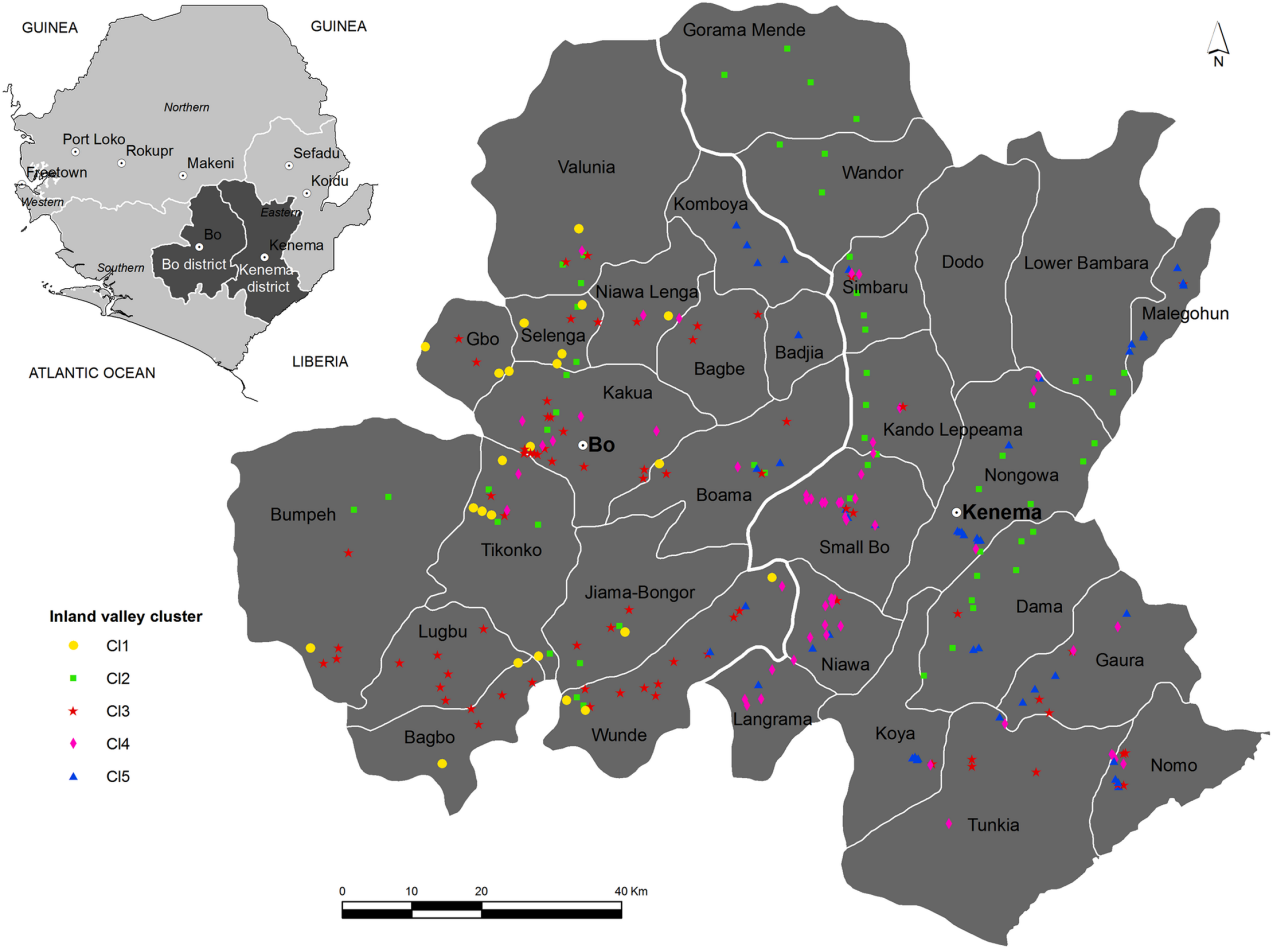
Location of the study area and inland valley cluster groups.

The present study was conducted on the inland valleys of the districts of Bo and Kenema (Fig 1), which represent the major regions in Sierra Leone where inland valleys occur [23]. The data was collected by the Rokupr Agricultural Research Centre of the Sierra Leone Agricultural Research Institute. No specific permission was required to collect the data because the inland valleys were not in protected zone and the study did not pose any risk to individual privacy, animal or plant.

The climate in the study area as in the other regions of West Africa is determined by the interaction of two air masses with different moisture characteristics: (a) the maritime (humid) air mass originating from the Atlantic Ocean and associated with the south-western winds. This air mass is commonly referred to as the south-west monsoon; (b) the continental (dry) air mass originating from the African continent and associated with the north-eastern Harmattan winds (trade winds) [3]. The climate in the study area is tropical humid with two distinct seasons: the wet season from May to October and the dry season from November to April, each season lasting for about 6 months. Diurnal temperatures vary from 25 to 34°C although they could be as low as 16°C at night during the harmattan. The average monthly temperatures are around 26°C. The average annual rainfall is about 3000 mm. The rainfall pattern is unimodal and the wettest months are July and August. The heavy rains in the wet season usually result in high discharges and runoff which ranges from 20 to 40% of the total annual rainfall [24]. Rivers overflow their banks during this period, whereas in the dry season from November to March they are greatly reduced. The heavy rains and maritime influence lead to high humidity. Relative humidity is usually as high as 90% in the wet season and as low as 20% in the harmattan during the dry season.

In the study area (Bo and Kenema districts), as in the other districts of Sierra Leone, rice is the main staple food, eaten on a daily basis by almost every household. Rice is the most important food crop, widely grown by farmers across the country; other important food crops in Sierra Leone are cassava, sweet potato, maize, vegetables, millet, palm oil and groundnut [25]. Inland valley ecosystems account for about 20% of the total rice area in the country [26]. Livestock systems are largely dependent on the season, with free ranging during the dry season and grazing in the scrublands and other uncultivated lands during the wet season [27].

### Data collection and variables definition

Data were collected in two steps: an exploratory phase in March 2014 to select the study area and a field survey during the rainy seasons (May—October) of 2014 and 2015 to locate all the inland valleys and to collect qualitative and quantitative data (Table 1) based on questionnaires and informal interviews. A data collection unit consisted of a specific inland valley area with its corresponding group of users. Inland valleys were initially identified using topographic maps and Google Earth. Relatively low lines and potential streams necessary for water accumulation in lowland were identified with topographic maps. The contrast in terms of vegetation between lowland areas and their surrounding uplands was visualized with Google Earth, which provided an indication of the existence of inland valley. The location and size of inland valley were validated during the field survey. Data on morphological characteristics (size, average width and shape) were obtained from the field survey and from digital elevation maps, following the approach of Windmeijer and Andriesse [3]. Inland valley shape included transversal flat, transversal U or transversal V (Table 1). In total, 257 inland valleys were identified from a comprehensive inventory. These inland valleys were delineated using a global positioning system device and mapped using ArcGIS 10.2 ESRI (Environmental Systems Research Institute). Each inland valley was categorized, based on the dominant land use, as unused under natural vegetation; abandoned; or cropped.

**Table 1:** Description of themes and variables.

Theme	Scale type	Scale class
Variables		
**Physical characteristics**		
**District**	nominal	-
**Chiefdom**	nominal	-
**Inland valley size (ha)**	numeric	-
**Average width (m)**	numeric	-
**Cross-sectional shape**	nominal	U, V, flat
**Soil organic carbon, available phosphorus, total nitrogen, sand and clay (%), pH (H**_**2**_**O)**	numeric	-
**Hydrology**		
**Annual rainfall (mm)**	numeric	-
**Water source**	nominal	Rainfed, supplemental irrigation, irrigation only
**Flooding duration (week)**	numeric	-
**Duration of emerging water table (week)**	numeric	-
**Drainage / irrigation infrastructure**	nominal	No drainage, presence of canals for drainage and / or irrigation
**Land use**		
**Dominant land use**	nominal	Abandoned, cropped, natural vegetation
**Acreage of crops in rainy and dry seasons (ha)**	numeric	-
**Crop varieties**	nominal	Local, improved
**Mode of planting**	nominal	Direct seeding, transplanting
**Soil fertility management**	nominal	No fertilizer, mineral and / or organic fertilizer
**Bunds**	nominal	No bunding, simple bunding, contour bunds
**Socio-economic characteristics**		
**Population density (inhabitants km**^**–2**^**), distance to the nearest road and distance to market (km)**	numeric	-
**Quality of road to market**	nominal	No road, path, dirt and paved road
**Land ownership**	nominal	Individual, family, village, state
**Land accessibility**	ordinal	Easy, medium, difficult
**User ethnic group**	nominal	Native, migrant
**Dominant gender**	nominal	Men, women
**Mode of exploitation**	nominal	Individual, collective
**Source of agricultural inputs**	ordinal	In the village, at < 25 km, 25–50 km, 51–100 km, > 100 km
**Production objectives**	nominal	Own household consumption, market, own household consumption and market

To identify the soil constraints to plant growth, the physical and chemical characteristics of surface soils (0–20 cm) were determined. Composite soil samples consisting of twenty five cores each of 32 mm diameter were taken along a diagonal of each inland valley. Sampling was made in January 2015. Soils were air-dried, ground and sieved (2 mm) prior to analysis. Soil samples were analyzed for organic carbon, available phosphorus, total nitrogen, pH (H_2_O) and particle size distribution following standard methods [28]. The particle size distribution was determined based on the Robinson pipette method. The soil pH was determined using a soil-to-water ratio of 1 to 2.5. The soil organic carbon content was determined by chromic acid digestion and the total nitrogen by Kjeldahl digestion. The available phosphorus content of the soil was determined using the Bray-1 method (0.025 M HCl + 0.03 M NH_4_F). The soil potassium was extracted with 1 M NH_4_-acetate and the content was determined by flame emission spectrophotometry. The scheme used for the interpretation of soil chemical characteristics followed the classification of Sys et al. [29].

The history of inland valley use, hydrological functioning and the importance of the inland valley to the local communities were assessed using rapid rural appraisal (RRA). The information was collected from small groups of 5 to 20 farmers for each inland valley, making a total of 257 RRA sessions during the survey. Information on the flooding and water table regime, duration of use, land and crop management practices and socio-economic characteristics was obtained from the users of the inland valleys.

Secondary data included rainfall, population density, physical accessibility of inland valley and market opportunity. Rainfall data were obtained from Africa Rainfall Climatology, Version 2 (ARC_2_) [30]. Data on population density were obtained from Gridded Population of the World, Version 4 (GPWv4) [31]. The legal and physical accessibility of each inland valley was defined based on land tenure (land ownership and access to land) and the ease with which the inland valley was physically accessible (distance from inland valley to the nearest road and road quality). Market opportunity was defined based on the distance from the inland valley to the nearest market and the roads quality between the inland valley and the market. In total, the dataset comprised 36 variables (nominal, ordinal and numerical) and was divided into four themes (physical characteristics, hydrology, land use and socio-economic characteristics) (Table 1).

### Data analysis

The 257 inland valleys were treated as independent sites for the statistical analyses. Tests of significance were conducted using R statistical software [32]. Bivariate analyses were carried out using χ^2^ and t-tests. Relationships between inland valley uses and socio-economic attributes as well as biophysical characteristics were analyzed using multivariate techniques in four steps. First, principal components analysis (PCA) was used to identify the cropping systems (major crops per cropping season and crop rotation) in each inland valley. Based on these results, inland valley farmers’ production systems were defined according to the use of external inputs (fertilizers). The resulting production systems were further refined into inland valley farm types by taking into account the farmers’ production objective (own household consumption; own household consumption and market; market). Second, a probability distribution of variable modalities [33] and multiple factor analysis [34] were carried out to select the variables that best discriminated between the sample of inland valleys. Third, cluster analysis was conducted with the selected variables to derive a typology of inland valleys by the hierarchical ascendant classification. Fourth, the dependent inland valley farm type variable was related to independent variables using Spearman’s non-parametric correlations and correspondence analysis to identify the biophysical and socio-economic factors that affect inland valley uses.

## Results

### Inventory and distribution of inland valleys

A total of 257 inland valleys were inventoried in 27 chiefdoms of the districts of Bo and Kenema in Sierra Leone. Variability in population density, physical accessibility and market opportunities was observed between chiefdoms and consequently between inventoried inland valleys (Table 2). The chiefdoms of Kakua and Nongowa had the highest population density and were located 1 km from the paved road and less than 10 km from the market, while the chiefdoms of Komboya and Langrama had the lowest population density and were located more than 20 km from the paved road and from the market. Variation in population density, physical accessibility and market opportunities translated into differences in land use patterns (Table 2). About 50% of the inland valleys used for agricultural production were located in relatively high population-density areas (more than 100 people km^-2^), close to the paved road and to the market (less than 10 km) while about 75% of the inland valleys under natural vegetation were located in relatively low population-density areas (fewer than 100 people km^-2^), far from the paved road and from the market (more than 10 km). However, location attributes alone did not explain the major inland valley use as about 40% of the inland valleys abandoned by farmers were located in relatively high population-density areas close to the main road and to the market (eg. Nongowa, Table 2). Flooding regime and soil fertility may contribute to the non-agricultural use of abandoned inland valleys.

**Table 2:** Population density, distance from inland valley to paved road, to market and distribution of the major inland valley use categories identified in the study area.

Chiefdom	Population density (inhabitants km^–2^)	Distance (km)		Inland valleys		
		to road	to market	cropped	abandoned	under natural vegetation
**Kakua**	556	1	4	21	0	0
**Nongowa**	387	1	9	11	10	0
**Boama**	162	1	9	6	2	0
**Lower Bambara**	142	1	9	2	2	0
**Tikonko**	132	2	7	5	4	4
**Lugbu**	127	16	42	5	0	0
**Simbaru**	122	28	4	4	4	0
**Malegohoun**	111	10	30	8	0	0
**Bagbe**	109	15	19	10	0	1
**Small Bo**	105	1	8	18	3	0
**Kando**	101	13	9	3	3	0
**Jiama**	85	4	13	4	2	0
**Dama**	70	6	16	3	8	0
**Selenga**	70	1	10	1	3	5
**Gorama**	66	16	13	3	0	1
**Niawa**	57	14	14	17	0	1
**Wandor**	56	33	19	0	3	0
**Gaura**	54	2	19	9	0	0
**Bumpeh**	49	6	13	4	2	1
**Gbo**	43	1	11	2	0	2
**Koya**	40	10	35	6	0	0
**Wunde**	33	9	16	13	2	3
**Tunkia**	33	4	7	6	0	0
**Valunia**	32	1	18	3	4	1
**Nomo**	30	14	32	12	0	0
**Langrama**	28	21	30	1	5	0
**Komboya**	27	24	22	4	0	0
**SED**	241	6	20			
**P value**	<0.001	<0.001	<0.001			

SED: Standard error of the difference

### Inland valley uses

The major land use in the inland valleys was determined by soil properties and hydrological regime (Table 3). Inland valleys abandoned by farmers had the lowest soil organic carbon (C): 1.5%, available phosphorus (P): 3.1 ppm, total nitrogen (N): 0.05% and clay content (7%) while inland valleys under natural vegetation had the highest soil organic carbon: 4.2%, available phosphorus: 10.2 ppm, total nitrogen: 0.10% and clay content (9%). In addition, 82% of the semi-permanently flooded inland valleys (898 ha) were abandoned or under natural vegetation while 97% of the seasonally flooded inland valleys (603 ha) were used for agricultural production. Therefore, inland valleys abandoned by farmers had a semi-permanent flooding regime and very low soil fertility while inland valleys under natural vegetation also had a semi-permanent flooding regime but high soil fertility.

**Table 3:** Effects of hydrological regime and land use on inland valley soil properties.

Flooding regime	Use	Sample size (n)	Soil C (%)	Soil P (ppm)	Soil N (%)	Sand (%)	Clay (%)
**Semi-permanently** **flooded**							
	**Abandoned**	45	1.5	3.1	0.05	83	7
	**Cropped**	13	3.0	9.0	0.10	83	7
	**Under natural vegetation**	14	4.4	10.3	0.10	69	10
	**SED (Use type)**		0.3	2.6	0.006	1.8	0.6
	**Use type (P value)**		<0.001	<0.001	<0.001	<0.001	<0.001
**Seasonally flooded**							
	**Cropped**	180	3.7	9.0	0.10	71	9
	**Under natural vegetation**	5	3.8	10.0	0.10	71	8
	**SED (Use type)**		0.5	1.9	0.005	0.9	0.3
	**Use type (P value)**		ns	ns	ns	ns	ns
	**Significance (P value)**						
	**Flooding regime (FR)**		<0.001	<0.001	<0.001	<0.001	<0.001
	**Use type (U)**		<0.001	<0.001	<0.001	<0.001	<0.001
	**Interaction FR x U**		0.007	ns	ns	0.003	0.03

SED: Standard error of the difference; ns: not significant.

### Characterization of inland valley cropping systems and inland valley farm typology

**Fig 2:**
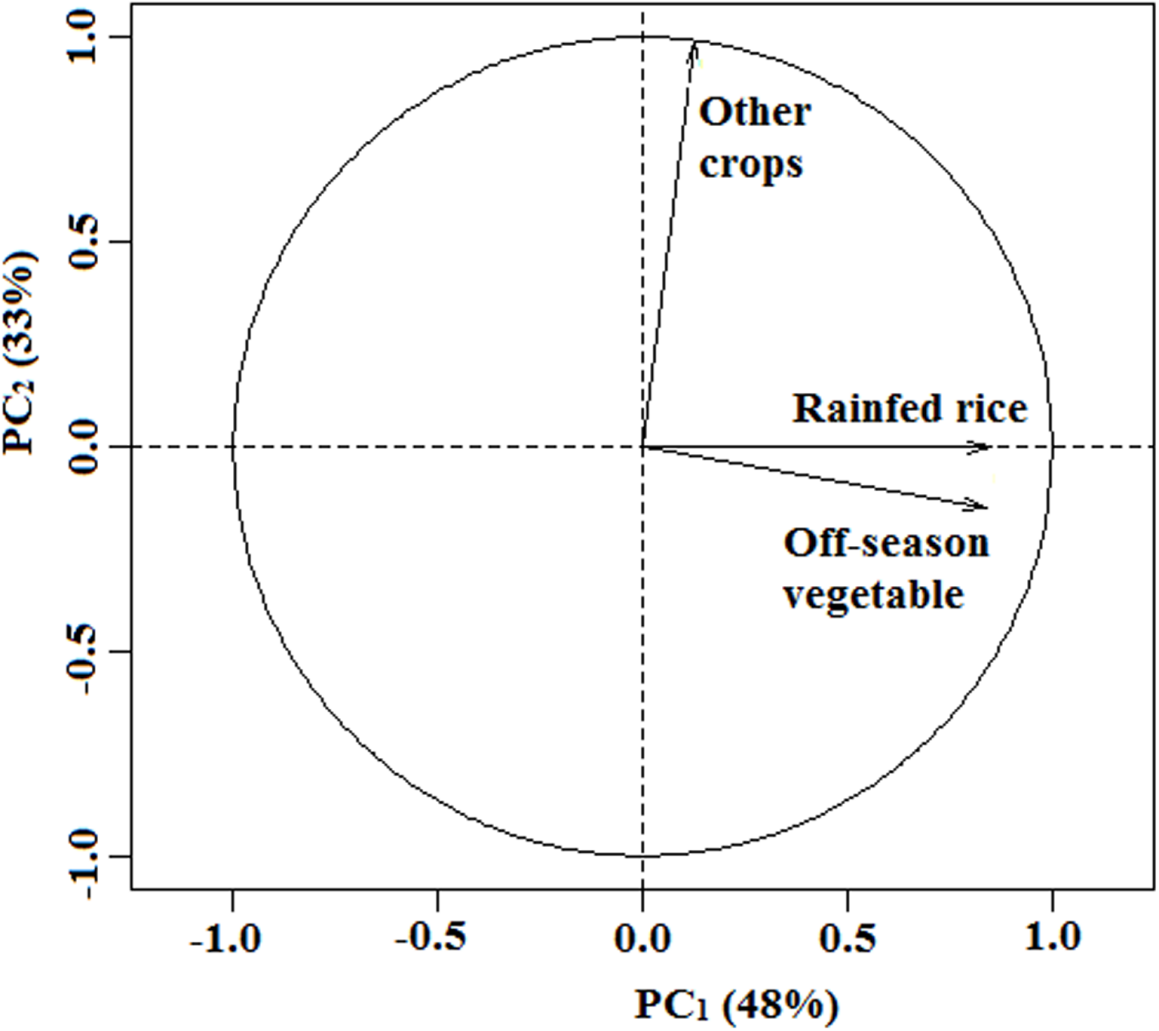
Projection of crop area on the first two axes of the principal components analysis.

**Fig 3:**
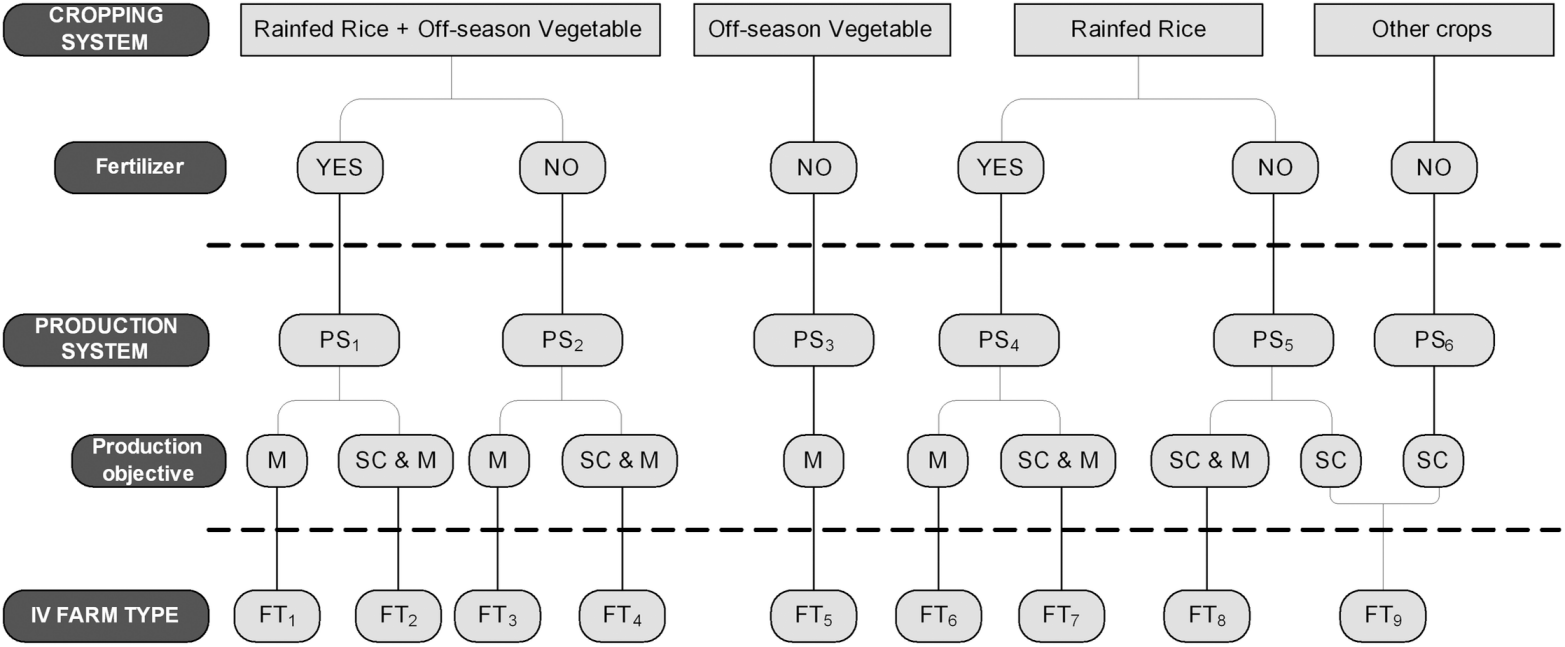
Cropping systems, farmers’ production systems and derived inland valley farm types in the agricultural inland valleys of the study area. Production objectives are market (M), subsistence (SC) or both (SC&M).

In the PCA on the area of crops produced in the inland valleys, the first two principal components (PCs) explained 81% of the total variance (Fig 2). Rainfed rice and off-season vegetables dominated PC_1_ with loading values up to 84%. PC_2_ was associated with other crops (cassava and maize) produced in the rainy season with loading value up to 98%. Four major cropping systems were defined based on this information: (i) only rainfed rice (34%; n = 88); (ii) rainfed rice and off-season vegetables (31%; n = 80); (iii) only off-season vegetables (6%; n = 15); and (iv) other crops (4%; n = 10). These four cropping systems were refined into 11 inland valley farm types by taking into account abandoned inland valleys, inland valleys under natural vegetation, and differences in soil fertility management (fertilizer application vs. no fertilizer application) and production objective (own household consumption; own household consumption and market; market) in the agricultural inland valleys (Fig 3; Table 4).

**Table 4:** Inland valley farm types defined by the study.

Inland valley farm type	Cropping system	Fertilizer application	Produced for
**FT**_**1**_	rainfed rice and off-season vegetables	with	market
**FT**_**2**_	rainfed rice and off-season vegetables	with	own household and the market
**FT**_**3**_	rainfed rice and off-season vegetables	without	market
**FT**_**4**_	rainfed rice and off-season vegetables	without	own household and the market
**FT**_**5**_	off-season vegetables	without	market
**FT**_**6**_	rainfed rice	with	market
**FT**_**7**_	rainfed rice	with	own household and the market
**FT**_**8**_	rainfed rice	without	own household and the market
**FT**_**9**_	subsistence farming: maize, cassava or rice	without	own household
**FT**_**10**_	inland valley under natural vegetation		
**FT**_**11**_	abandoned inland valley		

### Categorization of inland valleys

#### Variables selection

**Fig 4:**
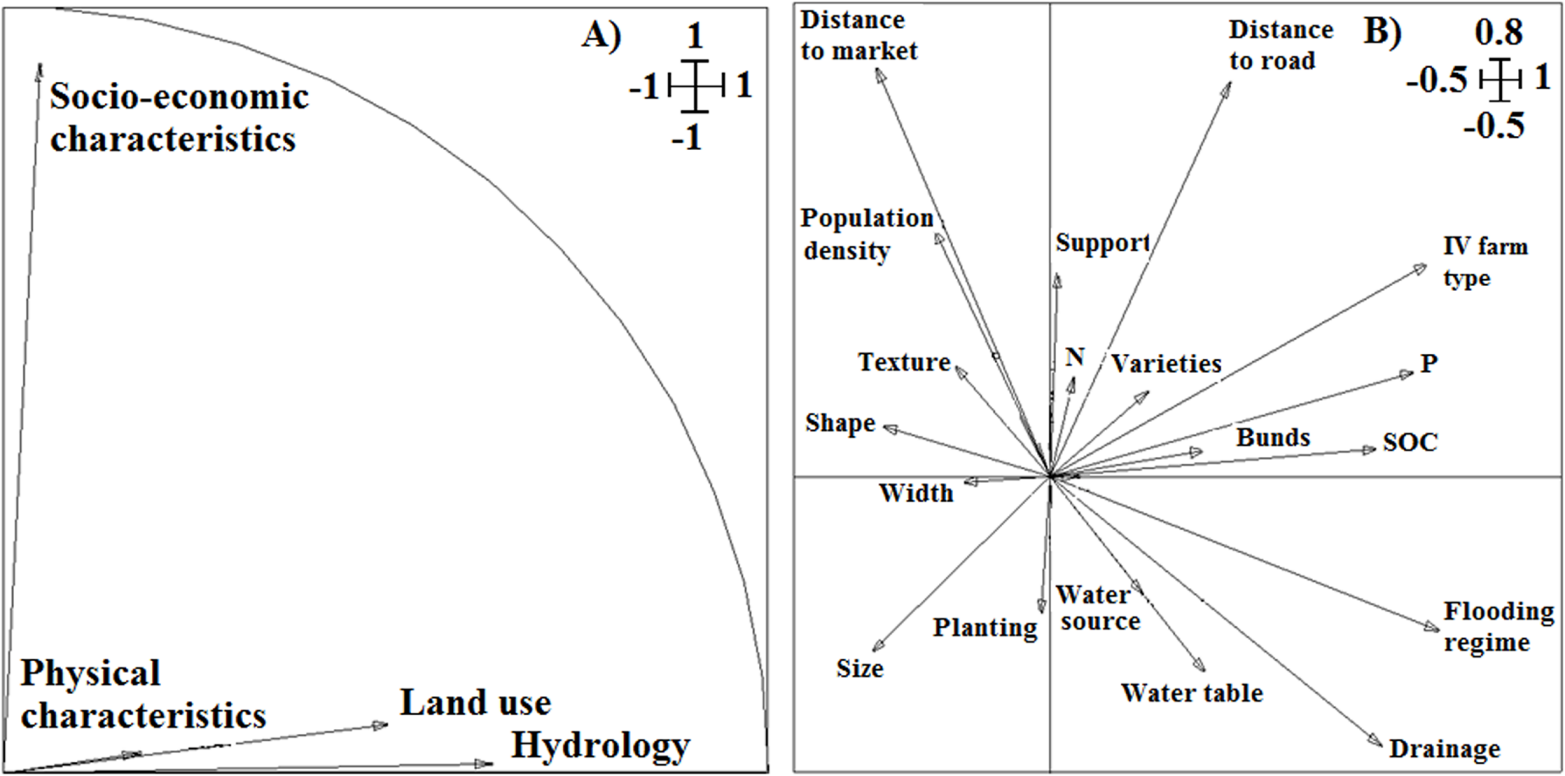
Projection of themes (A) and variables (B) on the factorial axes 1 x 2 of the multiple factorial analyses.

Some variables presented common modalities for the inland valleys surveyed. Such variables (rainfall, land ownership, users’ ethnic group and gender, mode of exploitation and source of agricultural inputs) were removed from the data set used to realize the multiple factorial analyses (MFA). The first four axes of the MFA explained 62% of the variation of the biophysical, socio-economic and land-use attributes within the dataset. The first axis explained 26% of the variability and was composed of the themes physical characteristics, hydrological regime and land use. The second axis explained 14% of the variability within the dataset and was composed of the theme socio-economic characteristics (Fig 4).The key variables that significantly differentiated the sample of inland valleys were flooding regime, drainage system, soil organic carbon, available P, and inland valley farm types, size and shape, which were correlated to the first axis; and distance from inland valley to the main road, to the market, and population density, which were correlated to the second axis (Fig 4), suggesting their importance as drivers of inland valley typology.

#### Inland valley typology

Five major inland valley clusters were identified using hierarchical cluster analysis (Fig 1).These comprised: (i) semi-permanently flooded inland valleys with high soil organic carbon (4.2%) and moderate available phosphorus (10.2 ppm), mostly under natural vegetation (9%, n = 23); (ii) semi-permanently flooded inland valleys with low soil organic carbon (1.5%) and very low available phosphorus (3.1 ppm), abandoned by farmers (22%, n = 57); (iii) seasonally flooded inland valleys with moderate soil organic carbon (3.1%) and low available phosphorus (8.3 ppm) used for rice and vegetables, produced without fertilizer application for own household and market (29%, n = 74), (iv) well drained inland valleys with moderate soil organic carbon (3.8%) and moderate available phosphorus (10.0 ppm) used for rice and vegetables, produced with fertilizer application for own household and market (21%, n = 55); and (v) well drained inland valleys with moderate soil organic carbon (3.6%) and moderate available phosphorus (11 ppm), produced without fertilizer application for own household (19%, n = 48).

#### Characterization of inland valley clusters

The main distinctive characteristics of the inland valley clusters are presented in Tables 5 and 6. Cluster 1 comprised semi-permanently flooded inland valleys mostly under natural vegetation. These included small (average size 2.1 ha) and concave inland valleys. Water covered the land surface throughout the growing season in most years. Of these inland valleys, 83% were covered by natural vegetation (FT_10_). The other 17% were cultivated only during the dry season, growing vegetables for market (FT_5_). The topsoil of the inland valleys of this cluster group showed the highest level of carbon (4.2%) and available phosphorus (10.2 ppm). These inland valleys were mostly located in areas far from the paved road (distance inland valley to paved road >10 km) with low population density (fewer than 100 inhabitants km^-2^) and poor market accessibility (distance inland valley to market > 20 km over paths and dirt roads).

**Table 5:** Ecological characteristics of inland valley clusters identified in the study area.

Parameter	Cluster 1	Cluster 2	Cluster 3	Cluster 4	Cluster 5
**Sample size**	23	57	74	55	48
**Morphology**	concave	concave	concave	flat	concave
**Inland valley size (ha)**	2.1±0.3	16.0±3	3.2±0.7	3.4±0.5	3.1±0.4
**Hydrology**	Water covers land surface throughout growing season in most years.	As cluster 1	Water covers land surface early in growing season, but is absent by end of season in most years.	Water covers land surface for brief periods during growing season, but water table usually lies well below surface for most of season.	As cluster 4
**Texture**	sandy loam	loamy sand	sandy loam	sandy loam	sandy loam
**Soil organic carbon (%)**	4.2±0.3	1.5±0.1	3.1±0.09	3.8±0.10	3.6±0.09
**Phosphorus (ppm)**	10.2±0.4	3.1±0.2	8.3±0.73	10.0±0.90	11±0.60
**Total nitrogen (%)**	0.10±0.01	0.05±0.01	0.1±0.01	0.1±0.01	0.1±0.01
Inland valley farm type*	FT_10_ (83%), FT_5_ (17%)	FT_11_ (100%)	FT_4_ (32%), FT_3_ (20%)	FT_2_ (31%), FT_1_ (16%)	FT_9_ (35%), FT_8_ (26%)

* See Table 4 for description of inland valley farm types

**Table 6:** Socio-economic attributes of inland valley clusters identified in the study area.

Parameter	Cluster 1	Cluster 2	Cluster 3	Cluster 4	Cluster 5
**Distance inland valley to road (km)**	13–15	10–13	6–8	6–9	10–14
**Distance inland valley to market (km)**	23–26	20–26	10–15	12–16	18–22
**Type of road from inland valley to market**	path (56%), dirt road (44%)	path (52%), dirt road (42%)	dirt road (44%), path (34%)	dirt road (45%), path (30%)	dirt road (58%), path (27%)
**Population density (inhabitants km**^**-2**^**)**	76–94	86–96	250–400	280–500	78–92

Cluster 2 comprised inland valleys abandoned by farmers. These included large (average size 16.0 ha) and concave inland valleys. Small sections of these inland valleys had been used in the past for subsistence crop production and then abandoned due to their coarse-texture topsoil (loamy sand), low soil organic carbon (1.5%), very low available phosphorus (3.1 ppm) and semi-permanent flooding regime. These inland valleys were mostly located in areas far from the paved road (distance inland valley to paved road >10 km) with low population density (fewer than 100 inhabitants km^-2^) and poor market accessibility (distance inland valley to market > 20 km over paths and dirt roads).

Cluster 3 comprised inland valleys seasonally flooded by stream overflowing or by run-off. Water covered the land surface for extended periods, especially early in the growing season, but was absent by the end of the season in most years. With a concave landscape, these inland valleys were small (average size 3.2 ha) and presented relatively low soil nutrient contents, with a mean value of 3.1% organic carbon and 8.3 ppm available phosphorus. These inland valleys were dominated by FT_3_ (20%) and FT_4_ (32%), mostly cultivated during the rainy season for rice and during the dry season for vegetables for the market and/or own household consumption without fertilizer application. Inland valleys of this cluster group were located in areas close to the paved road (distance inland valley to paved road < 10 km) with high population density (250–400 inhabitants km^-2^) and moderate market accessibility (distance inland valley to market 10–15 km over dirt roads and paths).

Cluster 4 comprised well drained inland valleys used for rice and vegetable produced with fertilizer application. Water covered the land surface for brief periods during the growing season, but the water table usually lies well below the surface for most of the season. With a flat landscape and small size (3.4 ha), these inland valleys were dominated by FT_1_ (16%) and FT_2_ (31%), mostly cultivated for rainfed rice and off-season vegetables with an application of organic and/or mineral fertilizers for the market and/or own household. Nutrient management in these inland valleys translated into moderate soil organic carbon (3.8%) and available phosphorus (10 ppm) content. These inland valleys were mostly located in areas closed to the paved road (distance inland valley to paved road < 10 km) with high population density (280–500 people km^-2^) and moderate market accessibility (distance inland valley to market: 12–16 km over dirt roads and paths).

Cluster 5 comprised well drained inland valleys used for subsistence crop produced without fertilizer application. Inland valleys of this cluster group presented similar hydrological regime like those of cluster 4. They were small (average size 3.1 ha), concave and dominated by FT_8_ (26%) and FT_9_ (35%), cultivated for maize, cassava or rice without fertilizer use produced for own household. The topsoil of these inland valleys showed a moderate level of soil organic carbon (3.6%) and available phosphorus (11 ppm). These inland valleys were mostly located in areas far from the paved road (distance inland valley to paved road > 10 km) with low population density (fewer than 100 people km^-2^) and moderate market accessibility (distance inland valley to market: 18–22 km over dirt roads).

### Inland valley–Inland valley farm type relationship

The 11 inland valley farm types were related to inland valley characteristics to provide insights into the factors affecting farmers’ decisions and strategies. Spearman’s correlations between inland valley farm type and the biophysical and socio-economic attributes of inland valleys revealed significant positive correlations between inland valley farm types and population density (r = 0.69, *P* = 0.01), market proximity (r = 0.60, *P* = 0.03), soil fertility (r = 0.50, *P* = 0.03) and accessibility (r = 0.50, *P* = 0.04). However, a significant negative correlation (r = 0.55, *P* = 0.02) was found for flooding regime. A weak and non-significant correlation was found between inland valley farm type and inland valley shape (r = 0.49, *P* = 0.14) and size (r = 0.44, *P* = 0.15).

**Fig 5:**
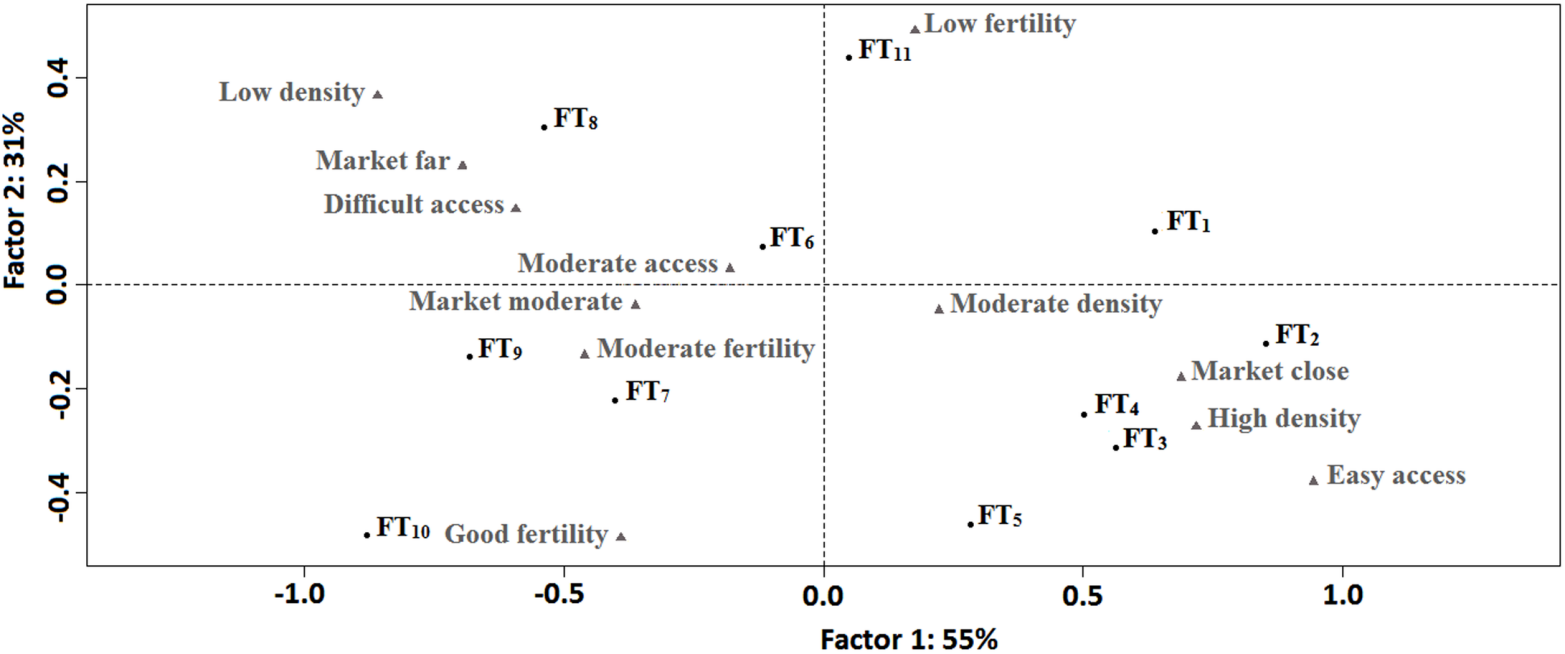
Positioning of inland valley farm types and inland valley characteristics on the first two axes of the correspondence analysis. Inland valley farm types are presented in black, descriptive variables in gray.

Based on these variables that positively correlated with farming in the inland valleys, correspondence analysis was used to describe the inland valley farm types under different biophysical and socio-economic conditions (Fig 5). The first axis explained 57% and the second axis 23% of the total variance in the dataset of biophysical and socio-economic characteristics of inland valleys and inland valley farm types. Axis 1 discriminated positively the inland valley farm types with two cropping seasons (rainfed rice and off-season vegetable) per year (FT_1_, FT_2_, FT_3_, FT_4_); and negatively the inland valley farm types with one cropping season per year without fertilizer use (FT_8_, FT_9_) and the uncultivated inland valleys under natural vegetation (FT_10_) based primarily on population density, distance of inland valley to market and inland valley accessibility. Axis 2 discriminated positively the abandoned inland valleys (FT_11_) and negatively the inland valley farm type with only off-season vegetable production for market (FT_5_) and the uncultivated inland valleys under natural vegetation (FT_10_) based primarily on soil fertility. Consequently, inland valleys intensively cropped by farmers (rainfed rice + off-season vegetable) were easily accessible, close to the market and found in high population-density areas, while uncultivated inland valleys under natural vegetation and inland valleys cropped only under rainfed conditions without fertilizer use were found in remote areas with poor market accessibility and low population density. Furthermore, inland valleys cropped for vegetables only in the dry season due to flooding during the rainy season, and uncultivated inland valleys under natural vegetation, had relatively higher soil fertility, while abandoned inland valleys had very low soil fertility. These results suggest that opening up the areas in which inland valleys occur through improved roads and markets, and better water control through drainage infrastructures, would promote crop diversification and intensification in the relatively little farmed inland valleys (cluster 1). These results also confirm the low potential for rice-based-cropping systems in inland valleys abandoned by farmers (cluster 2) due to their very low soil fertility and difficult flooding regime.

## Discussion

The typology developed in this study combined rural and participatory approaches, spatial and multivariate analysis to unscramble the complexity in heterogeneous inland valley systems and better understand their agricultural use. The use of different approaches for data collection and analysis is important to relate the inland valleys’ biophysical and socio-economic characteristics to the decisions of farmers who live in the surrounding environment and contribute to understanding the functioning of the inland valleys, which is critical for their sustainable use. Similar approaches have been used to classify the inland valleys in East Africa [15, 35].

The inland valley farm type is linked, either directly or indirectly, to population density and the subsequent land scarcity and market opportunity. These factors drive agricultural land use [36, 37] and are reflected in the inland valley cluster groups. Population growth has often led to land shortage in Sierra Leone [38] and hence has increased the need for agricultural production land. This has resulted in the expansion of cropland to inland valleys where inland valleys are accessible [39]. Good market access favors potential for farm inputs (fertilizer, improved seeds and agrochemicals) and farm outputs (market-oriented rice and vegetables), which are common features of agricultural intensification. Market opportunity and rural population density are frequently correlated and their effects on inland valleys uses have been reported in several studies [15, 40, 41]. Population growth and subsequent land shortages of arable land coupled with good market opportunity can partly explain the continuous crop production in inland valleys of clusters 3 and 4. However, crop diversification and intensification systems dominate the inland valleys located in high population-density areas with good market opportunities (clusters 3 and 4), whereas traditional farming systems are concentrated in inland valleys located in remote areas with low population density (cluster 5). The price at which farmers sell agricultural products and the contribution of each major crop to the household revenue could contribute to explain the diverse uses of inland valleys.

Farmers’ production objectives in conjunction with market access are likely to increase the conversion of inland valleys for food and production of high-value crops. This supports the capacity of inland valleys for diversified uses [35], but also suggests an increase in land use intensity as in cluster groups 3 and 4 in our study. Consequently, transitions between inland valley cluster groups through the corresponding inland valley farm types can be expected in the future with changes in market opportunities and population density. The study suggests that such drivers, in combination with free access rights to land in the inland valley, will exacerbate the pressure on inland valley production resources.

A combination of factors, including the growing food demand from urban centers [42] and the potential for income generation in inland valleys [43] are expected to increase the market orientation of inland valley production activities. This may result in increased land-use intensity of inland valleys through several seasons of market-oriented high-value crop production. Such land-use intensification contributes to livelihood diversification and hence to overall food and nutrition security.

Inland valleys have been used for crop farming partly because of their inherent high production potential [4]. This potential for crop production depends on the interplay between factors including climate, soil types and hydrology [44], which enables the functioning of the inland valley ecosystems, performing various socio-economic functions for different user groups [35]. This is reflected in the current study, where the agricultural use of inland valleys contributes to the livelihood (own household consumption and cash) of rural households.

However, inland valley cultivation has shown negative effects on soil fertility. Intensive cultivation of inland valleys without application of organic and mineral fertilizers has resulted in declining soil fertility. This is observed in the variability of soil fertility indicators between intensively cultivated inland valleys (cluster 4: fertilizer use, higher soil fertility and cluster 3: no fertilizer use, lower soil fertility), compared with cluster 5 (only rainfed rice cultivation, without fertilizer use, higher soil fertility). Therefore, intensive crop production under low external input levels can be detrimental for the soil and undermine long-term crop production [45]. Inland valleys of cluster 2 showed very low potential for rice-based cropping systems and have been abandoned by farmers as consequence of their very low soil fertility, particularly their severe limitation in available phosphorus. Thus, the typology revealed different categories of inland valley suitability for agricultural production under the diverse production systems used by farmers. In addition, the results revealed the importance of integrated nutrient management to replenish nutrient losses and sustain crop production as well as the development of road, market and drainage infrastructures for better capitalization of high-potential inland valleys for rice-based systems (clusters 1, 3, 4, 5).

The proposed typology considers land uses and production potential of inland valleys under different socio-economic environments and managerial decisions to identify specific conditions of inland valley uses. Each cluster group of inland valleys identified by the study combines biophysical conditions, socio-economic circumstances where inland valleys occur and farmers’ agricultural practices and production objectives providing a more comprehensive socio-ecological classification of inland valleys than those in prior research in sub-Saharan Africa. Various frameworks that have been developed for inland valley classification in sub-Saharan Africa included: those primarily based on biophysical characteristics [3, 16, 17, 18, 19] and those based on biophysical and socio-economic characteristics [20, 21, 15]. The proposed typology went beyond the existing frameworks and included farmers production systems and production objectives. The typology can be used to guide the selection of representative inland valleys for multi-disciplinary in-depth studies and the implementation of appropriate actions taking into account the drivers and decision factors of inland valleys-dependent communities.

## Conclusions

This study has contributed to unravelling the diversity of inland valleys by combining their physical, hydrological, land-use and socio-economic attributes. Five cluster groups of inland valleys were identified based on the hydrological conditions, soil characteristics, population density, market opportunity and inland valley farm types. The derived inland valley cluster groups and associated farm types were linked to the inland valley environment, relating the land user to the prevailing land-use factors (use type and use intensity) and biophysical characteristics of the inland valley. Such associations revealed the interactions between decision making units and their heterogeneous environment, which can be used to analyze and explore changes and dynamics in inland valley use. The analyses presented in this study can provide a framework for a comprehensive assessment of inland valleys diversity and a tool for targeting technologies intervention.

## Supporting information

S1 Fig Location of inland valley cluster groups. (CSV)

S2 Fig Crop area. (CSV)

S3 Fig Inland valley farm types. (CSV)

S4 Fig Themes and variables. (CSV)

S5 Fig Inland valley farm types and inland valley characteristics. (CSV)

S1 Table Description of themes and variables. (CSV)

S2 Table Population density, distance from inland valley to paved road, to market and distribution of the major inland valley use categories identified in the study area. (CSV)

S3 Table Effects of hydrological regime and land use on inland valley soil properties. (CSV)

S4 Table Inland valley farm types defined by the study. (CSV)

S5 Table Ecological characteristics of inland valley clusters identified in the study area. (CSV)
